# Individual alpha peak frequency is slower in schizophrenia and related to deficits in visual perception and cognition

**DOI:** 10.1038/s41598-021-97303-6

**Published:** 2021-09-08

**Authors:** Ian S. Ramsay, Peter A. Lynn, Brandon Schermitzler, Scott R. Sponheim

**Affiliations:** 1 Department of Psychiatry & Behavioral Sciences, University of Minnesota, Minneapolis, MN USA; 2 Minneapolis Veterans Affairs Health Care System, Minneapolis, MN USA

**Keywords:** Attention, Intelligence, Schizophrenia, Neuroscience, Neuronal physiology

## Abstract

The brain at rest generates cycles of electrical activity that have been shown to be abnormal in people with schizophrenia. The alpha rhythm (~ 10 Hz) is the dominant resting state electrical cycle and each person has a propensity toward a particular frequency of oscillation for this rhythm. This individual alpha peak frequency (IAPF) is hypothesized to be central to visual perceptual processes and may have downstream influences on cognitive functions such as attention, working memory, or problem solving. In the current study we sought to determine whether IAPF was slower in schizophrenia, and whether lower IAPF predicted deficits in visual perception and cognition that are often observed in schizophrenia. Eyes-closed resting state EEG activity, visual attention, and global cognitive functioning were assessed in individuals with schizophrenia (N = 104) and a group of healthy controls (N = 101). Compared to controls, the schizophrenia group showed slower IAPF and was associated with poorer discrimination of visual targets and nontargets on a computerized attention task, as well as impaired global cognition measured using neuropsychological tests across groups. Notably, disruptions in visual attention fully mediated the relationship between IAPF and global cognition across groups. The current findings demonstrate that slower alpha oscillatory cycling accounts for global cognitive deficits in schizophrenia by way of impairments in perceptual discrimination measured during a visual attention task.

## Introduction

Individuals with schizophrenia demonstrate abnormal oscillations in their brain activity when measured during rest^1–5^. Specifically, there is evidence that aberrant alpha rhythms may underlie distinct aspects of psychosis-spectrum psychopathology^3,6–8^ and relate to disrupted cognitive processes^9,10^. Alpha oscillations (~ 10 Hz) are consistently identified as a “dominant” frequency in human electroencephalogram (EEG) and most reliably observable in recordings from a person in an eyes-closed, relaxed, and awake state. While historically thought to reflect the “idling brain,” recent investigation supports a more nuanced role of alpha, wherein ~ 10 Hz rhythms are crucial for inhibition and gating processes that allow brain regions to effectively communicate while also suppressing activity irrelevant to task demands^11^. These relationships have been demonstrated during rest, where increases in posterior alpha power corresponded to reduced occipital and thalamocortical connectivity^12^, implying that alpha rhythm timing and power may be a manifestation of long-range cortical and subcortical communication.

Individual alpha peak frequency (IAPF) is defined as the frequency of the strongest alpha oscillation observed by electroencephalography (EEG) during rest, and is prominent over occipital and parietal brain regions when the eyes are closed^13^. IAPF holds unique relevance for visual perception and information processing, as animal research has demonstrated that inhibitory firing of thalamocortical neurons in the lateral geniculate nucleus is temporally framed by the positive and negative phase of the alpha cycle^14^. Similarly, both pre-stimulus alpha amplitude and phase have been shown to predict visual perception in humans^15^.

These findings in both animals and humans are consistent with the “inhibition-timing” hypothesis, which posits that the timing and phase of an inhibitory alpha rhythm is functionally related to the timing of neuronal activity more broadly^16,17^. Therefore, while reductions in alpha power may reflect the extent to which neural resources are devoted to a cognitive function (for example in instances of event-related desynchronization), IAPF may reflect oscillatory timing shared across neural populations and networks crucial for regulating sensory input^13^. In support of this, psychophysiological experiments have established that IAPF is distinctly associated with sensory perception, such that individuals with higher IAPF could distinguish between visual stimuli with shorter stimulus onset asynchronies^18^. Similarly, individuals with higher IAPF (and therefore faster alpha cycles) were susceptible to the cross-modal sound-induced flash illusion at shorter stimulus onset asynchronies, indicative of faster perceptual timing^19^. Relatedly, neuroimaging studies have demonstrated that higher IAPF is associated with increased regional cerebral blood flow in a network of brain areas crucial for attention^20^. In the current investigation we sought to characterize alpha oscillatory frequency abnormalities in schizophrenia and test their relevance to deficits in visual attention and cognition often observed in the disorder.

IAPF’s relationship with cognitive states has been demonstrated through behavioral experiments that have revealed shifts in IAPF during increased cognitive load and memory consolidation^21,22^, and has been shown to be related to processing speed^23^, working memory^24,25^, and motor control^26^. Subsequently, IAPF measured at rest has been found to be related to global cognitive ability^27^, where higher peak frequencies during eyes opened and eyes closed conditions strongly correlated with a latent variable summarizing broad aspects of higher-order cognition. Despite shifts in response to cognitive demands, IAPF measured during rest has otherwise been found to have strong test–retest reliability^28^, be heritable^29,30^, and have remarkable stability even following an intensive cognitive training intervention^31^. Together, this literature suggests that IAPF may be state-dependent during cognitive effort and trait-like at rest, and serve as an index of the pace at which neuronal populations oscillate. Ultimately, the timing of this alpha oscillatory rhythm could influence the likelihood that sensory stimuli will be processed, and therefore support cognitive functions dependent on sensory processing.

Given consistently documented generalized cognitive deficits in schizophrenia^32^, it is perhaps not surprising that reduced IAPF is also observed in this population^33–37^. Notably, disruptions in visual perception are hypothesized to underlie these global cognitive disruptions^38–40^, and have influence on impaired functional outcomes in the disorder. This may be manifested at the most basic level of visual perceptual impairment, all the way up to disruptions in visual attention or higher-order representations that likely interact and contribute to aspects of cognitive impairment^41^. Thus, disturbances in IAPF measured during rest may account for disruptions in visual perception and higher-order cognition in schizophrenia. Clarification of these relationships could offer insights into the neural origins of global cognitive deficits in schizophrenia, as well as provide a plausible treatment target for cognitive, behavioral, and neuromodulatory interventions.

To understand the role of IAPF (measured between 7.25 and 13 Hz) in individuals with schizophrenia (SCZ), the current study examined eyes closed resting EEG in a sample of individuals with schizophrenia psychopathology (i.e. schizophrenia and schizoaffective disorder) compared to healthy control comparison subjects (CON). We focused on eyes closed resting EEG based on prior work that established strong test–retest reliability and modest relationships with cognition within this condition^31^. Despite previous work primarily examining alpha peak frequencies over posterior scalp locations^13^, recent findings indicate that alpha oscillations may function as a ‘traveling wave’ that reflects the passing of information across a hierarchy of cortical regions^42^ thus serving as a marker of cortical processing that is observable across the scalp. We therefore hypothesized that there would be group differences in this quantitative EEG measure across the scalp. To further clarify whether aberrant IAPF may account for disruptions in perception and cognition observed in SCZ^40^, we examined the relationship between IAPF and visual attention (measured using the Degraded-Stimulus Continuous Performance Test; DS-CPT) and global cognition (measured by subtests from the Wechsler Adult Intelligence Scale-III; WAIS-III). Consistent with evidence that oscillations in the alpha range drive perceptual rhythms critical for visual perception and higher-order cognitive processing processing^43^, we hypothesized that lower IAPF would reflect poorer target and nontarget discrimination (lower d′) on the DS-CPT (indicative of worse visual attention) as well as lower global cognition scores.

## Methods

### Participants

Individuals with schizophrenia (SCZ; N = 104) and healthy control subjects (CON; N = 101) ages 18–60 years old were recruited as part of four separate study protocols that implemented a measure of resting state EEG. Participants completed extensive diagnostic interviews by trained research staff using the Structured Clinical Interview for DSM-IV-TR (SCID) and the Diagnostic Interview for Genetic Studies (DIGS). Available clinical research materials were reviewed by two or more PhD-level clinicians or advanced graduate students to establish consensus diagnoses for each research participant. Individuals in the SCZ group were required to be clinically stable outpatients with a diagnosis of either schizophrenia or schizoaffective disorder, while healthy control subjects were required to have no psychotic or mood disorder (including bipolar disorder). Additionally, controls were required to have no first-degree relatives with psychotic or mood disorders. Any participant with alcohol or drug abuse in the past month or alcohol or drug dependence in the last 6 months was excluded. Participants were also excluded if they had compromised hearing or vision (i.e. unable to hear without a hearing aid or legally blind), had an estimated IQ < 70, a history of epilepsy or recurrent seizures in adulthood, or any other neurologic or medical condition that might preclude participation. N = 90 SCZ participants were on a stable dose of an antipsychotic medication, and N = 41 SCZ participants reported being employed in the prior month. Study procedures were approved by the institutional review boards of the University of Minnesota and the Minneapolis Veterans Affairs Medical Center, and all participants gave written informed consent prior to participation. All study procedures were performed in accordance with relevant guidelines and regulations.

Participants underwent the resting EEG procedures described below. A subset of participants (N = 181; SCZ = 95; CON = 86) also completed the Degraded Stimulus Continuous Performance Test (DS-CPT; Program for IBM-Compatible Micro Computers, Nuechterlein and Asarnow, 1999), which is a visual attention task that measures vigilance by requiring subjects to distinguish “target” from “non-target” stimuli where both the background and stimuli are visually degraded (see^44,45^ for more details). Briefly, numerical stimuli (0–9) were presented for 29 ms followed by a 971 ms white display. Stimuli were degraded such that 40% of white stimulus pixels were switched to black, and 40% of black background pixels were switched to white. Subjects were asked to respond with a button press to target trials (“0” stimuli making up 25% of trials) and withhold responses to all other stimuli (1–9 making up the remaining 75% of trials). Another subset of participants (N = 167; SCZ = 85; CON = 78) were administered four subtests from the Wechsler Adult Intelligence Scale-Third Edition (WAIS-III). These included ‘Block Design’ to measure perceptual reasoning, ‘Digit Span’ to measure working memory, ‘Digit-Symbol Coding’ to measure processing speed, and ‘Vocabulary’ to measure verbal reasoning. Raw subtest scores were converted to age-normed scaled-scores provided in the WAIS-III manual. Individuals in the SCZ group also underwent an evaluation of positive and negative psychosis symptoms using the Scale for Assessment of Positive Symptoms (SAPS)^46^ and the Scale for Assessment of Negative Symptoms (SANS)^47^.

### EEG acquisition

Three different EEG collection systems and montages were used across the four studies. N = 70 (SCZ = 29; CON = 41) recordings were obtained using a BioSemi 64-channel ActiveTwo AgCl electrode system, and N = 102 (SCZ = 51; CON = 51) recordings were obtained using a BioSemi 128-channel ActiveTwo AgCl electrode system (BioSemi Inc., Amsterdam, The Netherlands). In both cases, recordings were sampled at 1024 Hz and referenced after collection to an average ears signal. N = 37 (SCZ = 24; CON = 13) recordings were collected using a BrainVision 128-channel actiCHamp active electrode EEG system (Brain Products GmbH, Gilching, Germany) sampled at 1000 Hz and referenced online to Cz. In all data collection procedures, participants were asked to alternate between 45s periods of keeping their eyes open and keeping their eyes closed to ensure wakefulness. During eyes-open periods, they were instructed to attempt to minimize their blinking and to focus on a small fixation cross presented on a computer screen in front of them. Each state was repeated at least three times, for a minimum of 135 s of recorded EEG for both eyes-open and eyes-closed states. Only data from the eyes-closed period was used in the current analysis.

### EEG preprocessing

For all studies and systems, EEG processing was conducted using Matlab (The Mathworks, Inc., Natick, MA), including low-pass filtering (256 Hz cut-off frequency), high-pass filtering (0.5 Hz cut-off frequency), down-sampling to 256 Hz using an anti-aliasing resample function, and referencing to a linked earlobe signal. For preprocessing, data collected from BioSemi systems (N = 172) were referenced to a mean of the preauricular signals, while data collected from the BrainVision system (N = 37) was referenced to Cz. Data preprocessing was accomplished via a custom independent component analysis (ICA) based method for removal of ocular, muscular and cardiac artifact and spherical spline interpolation of bad electrode signals^48^; the method combines automated methods for excluding contaminated epochs with visual-inspection of final independent components to identify primarily brain-dominant versus artifact-dominant components. Denoised brain-dominant components were finally reconstituted to produce cleaned recordings for further analysis. Previous work has demonstrated that measures of spectral power from resting EEG are highly reliable from ~ 1 min of resting EEG data^49^, therefore only subjects with at least 1 min of artifact-free data were included in the current analysis. Across groups, subjects had an average of 119.80 s (SD = 21.49) of analyzed data with no statistical differences between groups (*p* = 0.45).

**Figure 1:**
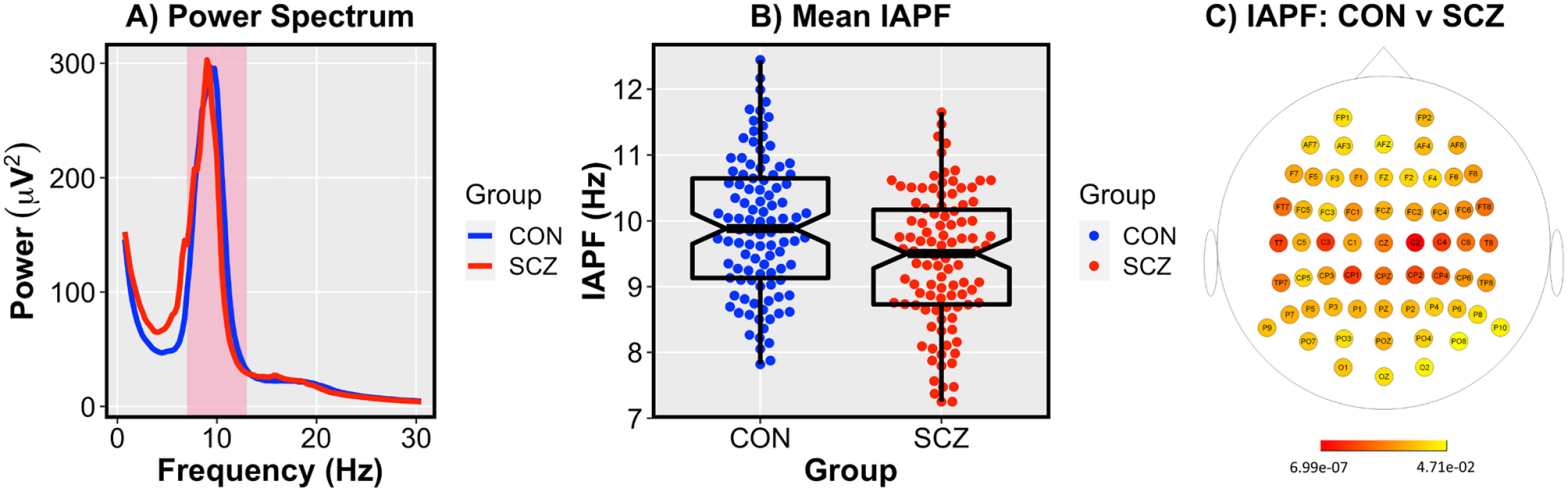
(**A**) Power spectrum from 0.5 to 30 Hz for CON (Blue) and SCZ (Red) groups (pink shaded area reflects 7.25–13 Hz range). A depiction of the log–log power spectrum is available in the supplemental material (Supplementary Fig. 1). (**B**) Mean IAPF across all electrodes was higher in CON (M = 9.91 Hz) compared to SCZ (M = 9.41 Hz; *t* = 3.71; *p* = 0.0003). Alternative depiction of the distribution of IAPF across groups is available in the supplemental material (Supplementary Fig. 2). (**C**) IAPF was lower in SCZ compared to CON across all electrode sites [FDR-corrected p < 0.05; colored electrodes reflect significance-level (log-transformed p-values)]. Topography of IAPF in the SCZ, CON, and averaged across groups is available in the supplemental material (Supplementary Fig. 3).

Denoised recordings were ultimately re-referenced to the average signal across all scalp electrodes. Thereafter, the continuous data was arbitrarily epoched into 4 s periods, and spectral power (in 0.25 Hz frequency bins) was computed via application of a discrete Fourier transform to each available electrode for each participant. Sixty-two 10–20 International System electrodes common to all systems (see Fig. 1C) were identified for each subject and used in the ensuing analyses. Additionally, we used the FOOOF (Fitting oscillations & one over f) toolbox^50^ in Matlab to characterize the slope of aperiodic 1/f frequency in each subject at each electrode individually.

### Measurement of IAPF

Following individual subject pre-processing, the power spectral density at each electrode for each individual subject was visually examined to ensure there was a clear power peak in the 7.25 to 13 Hz range. If multiple peaks were detected, our analytic strategy favored the peak frequency with higher spectral power. One subject in the SCZ group showed consistent peaks across electrodes in the theta band (4–7 Hz) and was subsequently removed from analysis, leaving the final sample of N = 204 (SCZ = 103; CON = 101). Next, for each subject, IAPF was measured at each electrode as the frequency with the greatest spectral power observed in the alpha range (7.25–13 Hz). We chose this broader than typical range of alpha as we anticipated that SCZ subjects may have slower peak frequencies. Furthermore, to ensure that group differences were not simply driven by a lower or upper limit effect, we also calculated the ɸ-frequency (‘phi’ frequency) by determining the median frequency in a 3 Hz band centered on the alpha peak, and therefore not explicitly bounded between 7.25 and 13 Hz^51^. The mean of the IAPF across all electrodes and the mean ɸ-frequency across all electrodes were strongly correlated (*r* = 0.94) and had strong intraclass correlation (ICC = 0.94), suggesting that the IAPF calculation was not biased by the upper (13 Hz) or lower (7.25 Hz) limit of the alpha frequency range.

### Planned analyses

We first compared IAPF between SCZ and CON by performing a one-way ANOVA at all 62 electrodes with covariates for age, gender, and study montage. We relied on a false-discovery rate (FDR) correction to identify individual electrodes that demonstrated a significant group difference (*p* < 0.05). Next, we performed a linear model (again with age, gender, and study montage as covariates) to determine whether mean IAPF predicted signal to noise discrimination (measured by d′—a signal detection measure comparing the scaled ratio of target ‘hits’ to ‘false alarms’) on the DS-CPT visual attention task. We then used a similar model (with age, gender, and study montage as covariates) to examine whether IAPF predicted global cognition (calculated as the mean age-normed scaled-score across the four WAIS-III subtests). To determine whether relationships between IAPF and global cognition were generalized or specific to cognitive sub-domains, we conducted independent post-hoc linear regressions on the processing speed, perceptual reasoning, working memory, and verbal reasoning subtests. Finally, to determine whether visual attention causally mediated the relationships between IAPF and global cognition, we used the ‘mediate’ package in R^52^. This package when applied to normally distributed datasets performs linear structural equation models similar to that described by Baron and Kenny^53^. N = 142 (SCZ = 77; CON = 65) subjects with global cognition, DS-CPT and IAPF data were available for the mediation analysis. We used a nonparametric bootstrapping procedure (10,000 permutations sampling with replacement) to obtain the average causal mediation effect (ACME) between IAPF and global cognition mediated by d’ on the DS-CPT. All figures were generated using R (R Core Team (2020). R: A language and environment for statistical computing. R Foundation for Statistical Computing, Vienna, Austria. https://www.R-project.org/).

## Results

The SCZ and CON groups did not differ on the basis of age or gender (*p’s* > 0.06) but did differ with regard to the global cognition score, the WAIS-III subtest domain scores, as well as d’ on the DS-CPT (see Table 1). The SCZ group showed reduced IAPF compared to the CON group over every electrode site (FDR-corrected *p* < 0.05; see Fig. 1C). These results remained significant when additionally covarying for the aperiodic slope of each electrode for every subject (FDR-corrected *p* < 0.05). Because group differences did not vary by electrode, we performed the ensuing analyses on the mean IAPF (SCZ = 9.41 Hz; CON = 9.91 Hz) across all electrodes, which also significantly differed between groups in an ANCOVA with covariates for age, gender, and study montage (*F* = 14.73; *p* = 0.0002; see Fig. 1A,B; Supplementary Table S1).

**Table 1:** Demographics.

	SCZ (N = 104)		CON (N = 101)	SD	t-value	p-value
	M	SD	M			
Age	42.21	10.94	44.95	11.23	− 1.77	0.08
Years of education	13.58	2.28	15.22	1.89	5.55	1.0 × 10^–7^
Global cognition (age-normed scaled score)	9.29	2.26	11.32	2.02	− 6.12	6.5 × 10^–9^
Processing speed (age-normed scaled score)	8.34	3.04	11.43	3.22	− 6.43	9.5 × 10^–10^
Perceptual reasoning (age-normed scaled score)	9.32	3.25	11.12	3.07	− 4.04	0.00008
Working memory (age-normed scaled score)	9.41	2.60	10.63	2.80	− 2.94	0.004
Verbal reasoning (age-normed scaled score)	9.58	3.49	11.95	3.01	− 5.28	7.0 × 10^–7^
DS-CPT (d′)	2.02	1.07	2.42	1.06	− 2.54	0.01
Age of PSYCHOSIS ONSET	22.66	7.84	–	–	–	–
SANS (total score)	32.27	18.26	–	–	–	–
SAPS (total score)	19.83	16.58	–	–	–	–
Chlorpromazine equivalent dosage	532.50	420.57	–	–	–	–

SCZ (Males = 75; Females = 29) and CON (Males = 60; Females = 41) did not statistically differ on the basis of gender (Χ^2^ = 3.14; p = 0.08).

*SCZ* Schizophrenia, *CON* Controls, *DS-CPT* Degraded-Stimulus Continuous Performance Test, *d′* D-prime, *SANS* Scale for the Assessment of Negative Symptoms, *SAPS* Scale for the Assessment of Positive Symptoms.

**Figure 2:**
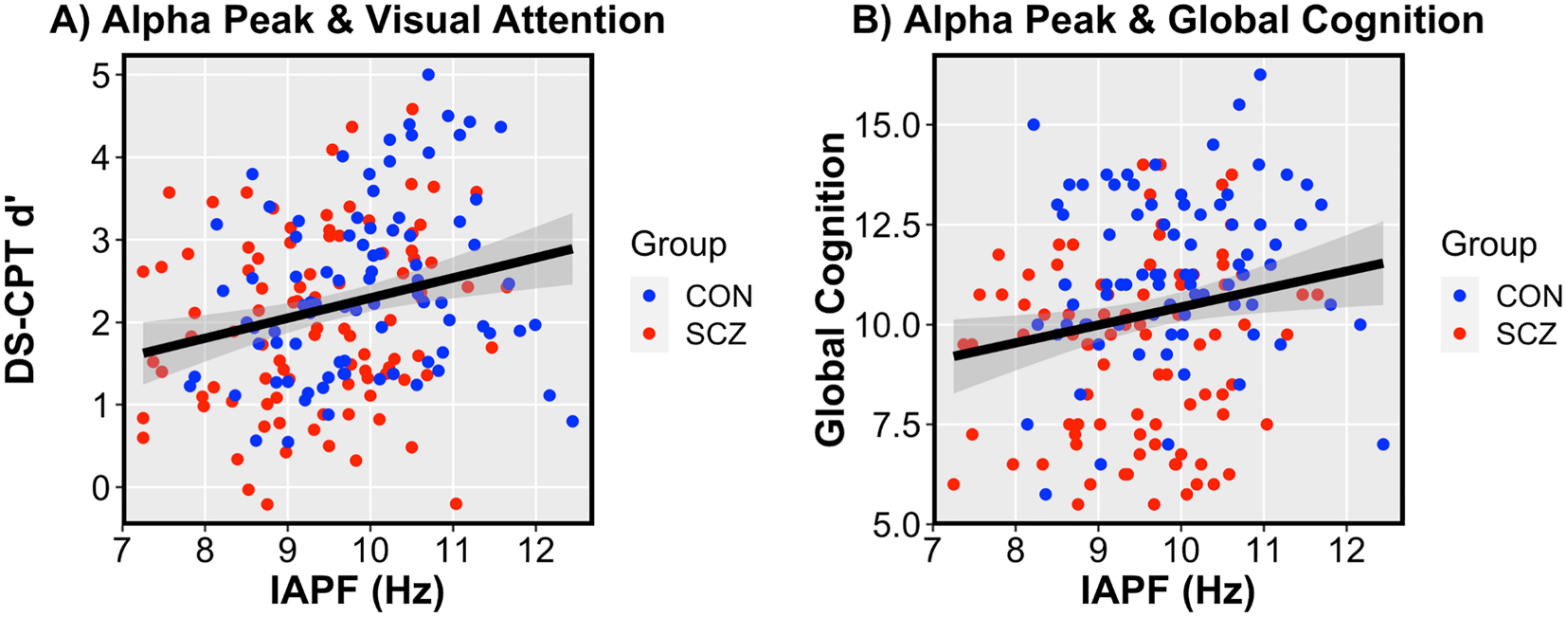
Mean IAPF was predictive of d′ on the DS-CPT and global cognition across both the CON and SCZ groups. (**A**) Mean IAPF predicted discrimination (d′) between targets and non-targets on a visual attention task (DS-CPT) across groups (*t* = 3.48; *p* = 0.0006). (**B**) Mean IAPF predicted global cognition measured on the WAIS-III across groups (*t* = 3.04; *p* = 0.003).

Next we used linear models (with covariates for age, gender, and study montage) to examine the relationships between IAPF and both discrimination (d’) on the visual attention task and global cognition. IAPF was predictive of d’ measured by the DS-CPT (*t* = 3.48; *p* = 0.0006; see Fig. 2A; Supplementary Table S2), suggesting that higher alpha frequencies are related to higher vigilance across groups as reflected in better discrimination between visually degraded target and nontarget stimuli. This relationship did not statistically differ between groups, but did appear to be more closely related to false-alarm rate (*t* = *− *3.38; *p* = 0.0009) as opposed to hit rate (*t* = *− *1.93; *p* = 0.055).

Similarly, IAPF was found to be predictive of global cognition (*t* = 3.04; *p* = 0.003; see Fig. 2B; Supplementary Table S3), indicating that higher individual alpha frequencies were related to stronger cognitive abilities. Again, this relationship did not differ between groups. We followed up on the cognitive subdomains to determine whether associations with IAPF were functionally specific. In separate post-hoc models, IAPF was predictive of processing speed (*t* = 2.94; *p* = 0.004; Supplementary Table S4), perceptual reasoning (*t* = 4.12; *p* = 0.00006; Supplementary Table S5), working memory (*t* = 2.23; *p* = 0.03; Supplementary Table S6), and verbal reasoning (*t* = 3.43; *p* = 0.0007; Supplementary Table S7), indicating that alpha frequency broadly related to cognition. These relationships did not differ between groups except for verbal reasoning (*t* = *− *2.21; *p* = 0.03), in which the CON group showed a significant positive relationship (*r* = 0.24; *p* = 0.01) while the SCZ group did not (*r* = *− *0.07; *p* = 0.46). No significant relationships were observed between IAPF and psychotic symptomatology as measured by either SANS or SAPS total scores in the SCZ group (*p’s* > 0.36). Additionally, IAPF showed no relationship to medication dosage measured in chlorpromazine (CPZ) equivalents in the SCZ group (*p* = 0.99), and no relationships between CPZ and DS-CPT d′, global cognition, or cognitive subdomain scores (all *p’s* > 0.41).

**Figure 3:**
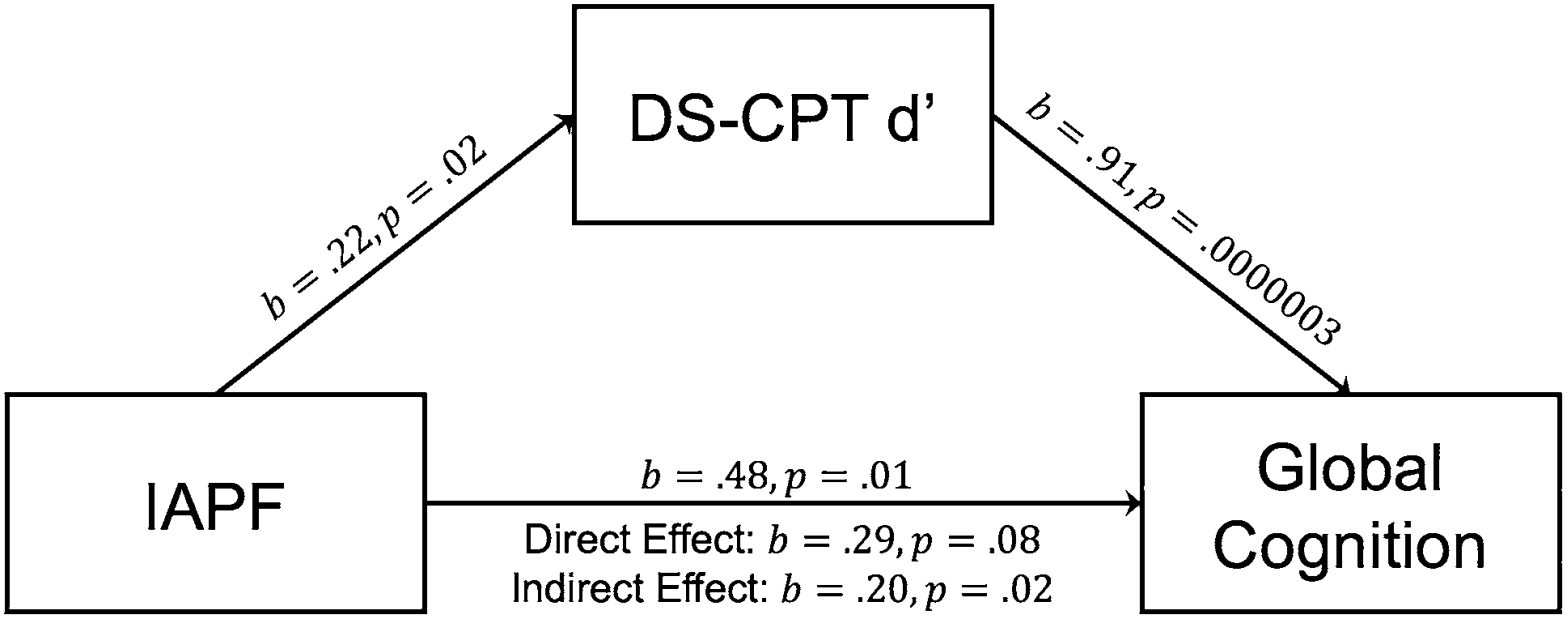
Target versus non-target discrimination (d′) on a visual attention task (DS-CPT) fully mediated the relationship between IAPF and global cognition across groups (Average Causal Mediation Effect (ACME) *b* = 0.20; 95% Confidence Interval 0.03–0.40; *p* = 0.02).

Given a strong relationship between global cognition and d’ on the DS-CPT (*t* = 3.35; *p* = 0.001), we sought to determine whether discrimination between targets and non-targets measured during this visual attention task mediated the relationship between IAPF and global cognition. The analysis demonstrated that d’ fully mediated the relationship between IAPF and global cognition across groups (Average Causal Mediation Effect (ACME) *b* = 0.20; 95% CI 0.03–0.40; *p* = 0.02), suggesting that deficits in stimulus discrimination during visual attention fully account for the relationship between lower IAPF and impairments in global cognition (Fig. 3). This mediation effect was not shown in the SCZ or CON groups independently, suggesting that this effect is driven by variation across the pathological and normative sample. To determine whether the mediation effect generalized across all cognitive domains measured, we performed post-hoc mediation analyses on the WAIS-III subtests independently. d′ consistently mediated the relationship between IAPF and processing speed (ACME *b* = 1.07; 95% CI 0.15–2.32; *p* = 0.02), perceptual reasoning (ACME *b* = 1.02; 95% CI 0.35–1.83; *p* = 0.002), working memory (ACME *b* = 0.19; 95% CI 0.02–0.47; *p* = 0.02), and verbal reasoning (ACME *b* = 0.86; 95% CI 0.24–1.70; *p* = 0.003), indicating that visual attention discrimination broadly drives the relationship between IAPF and a variety of cognitive functions.

## Discussion

Individual alpha peak frequency (IAPF) measured during rest with eyes closed was found to be reduced in schizophrenia (SCZ) compared to healthy controls (CON), suggesting that alpha oscillatory timing is broadly deviant in SCZ. Furthermore, slower IAPF was predictive of deficits in discrimination (d’) on a measure of visual attention (DS-CPT) as well as global cognition measured by the WAIS-III. Similar relationships were observed in specific domains of processing speed, perceptual reasoning, working memory, and verbal reasoning. These findings are consistent with findings in healthy adults demonstrating that the timing of alpha oscillations are predictive of the temporal width of visual perception windows^18^, as well as predictive of broader global cognitive abilities^27^. Findings of the current study provide evidence that slower IAPF may reflect a neural mechanism accounting for generalized cognitive impairment in schizophrenia.

Furthermore, we demonstrate that stimulus discrimination during visual attention fully mediated the relationship between IAPF and global cognition, offering evidence to further suggest that aberrant alpha rhythms drive global cognitive deficits via specific disturbances in visual perception. We propose that IAPF measured during resting EEG may in part reflect the level of perceptual sensitivity determined by the dominant frequency at which neuronal populations cycle in unison across broad regions of the brain. Our analysis suggests that such perceptual sensitivity ultimately plays a role in higher-order cognitive functions. Given previous animal and human neuroimaging findings establishing alpha peak’s role in the timing of excitatory and inhibitory processes^14,17^, the current findings highlight how the relative timing of dispersed neural activity is a plausible neural mechanism for generalized cognitive deficits in schizophrenia. It is also consistent with demonstrated thalamocortical disruptions observed in schizophrenia^54,55^, as work in animals and humans has found that alpha oscillations are dependent on the thalamus^56^, or may otherwise pace thalamocortical feedback loops^57^.

While the current findings are largely consistent with alpha’s observed role in sensory inhibition in the visual domain, it is less clear whether this may also extend to auditory perceptual processes. Indeed, the global cognitive deficits prominent in schizophrenia are likely driven by underlying disruptions in both visual and auditory processes^58^, though a common oscillatory mechanism to account for perceptual abnormalities across sensory systems has so far been elusive. Much like in visual perception, auditory signal-to-noise discrimination has been shown to reflect selective inhibition, but is driven by alpha oscillations generated in primary auditory cortex^59^. This further suggests that alpha oscillations are not exclusively generated in posterior occipital regions^60,61^, and may play a role in sensory inhibition more broadly. For example, experimental evidence has demonstrated that alpha oscillations can rhythmically bias and predict future auditory perception^62^, and is lateralized in response to directional (i.e. hemispheric) shifts in auditory attention^60^, both of which may have important implications for verbal working memory and attention. Alpha may therefore play an inhibitory role across sensory modalities^63^ and supports the notion that aberrant alpha rhythms may contribute to generalized cognitive deficits observed in schizophrenia. Future work will be required to test these hypotheses.

What also remains unclear is whether IAPF accounts for cognitive deficits in schizophrenia specifically, or characterizes a neuropathological mechanism that impacts sensory perception and cognition more broadly. IAPF has been found to reduce as a function of age^64^, is related to polymorphisms in the catechol-O-methyltransferase (COMT) gene^65^, and is accordingly hypothesized to account for aspects of cognitive decline. Reduced IAPF has also been observed in patients with dementia and Alzheimer’s disease^66,67^, in individuals on the autism spectrum (where fluctuations in IAPF correlated with cognitive functioning)^68,69^, observed in children with attention deficit disorder^70,71^, and related to symptoms of depression^72^. These observations across neuropsychiatric disorders and aging may indicate that alpha oscillatory timing broadly accounts for pathology related to cognitive impairment or decline.

In light of the current findings, it may be proposed that IAPF is a viable neural treatment target for interventions seeking to enhance cognition in schizophrenia (and potentially other disorders where neurocognitive decline is prominent). Previous studies have used neuromodulatory interventions (e.g. tES or TMS) to target IAPF both in schizophrenia^73^ and healthy individuals^74,75^, demonstrating changes in alpha power. In healthy adults ‘online’ neuromodulation (i.e. during task engagement) using transcranial alternating current stimulation (tACS) above or below IAPF was shown to modulate visual perception at either narrower or wider stimulus onset asynchronies^76^. This finding offers definitive evidence for the individual alpha peak reflecting the gating of temporal windows critical for accurate visuo-perceptual processing, indicating not only that the timing of IAPF plays a causal role in perception, but that it can be systematically enhanced with non-invasive neuromodulation. However, there is little evidence that such neuromodulation techniques result in sustained changes in IAPF measured during rest^77^ (though also see^78^), and may quickly return to a baseline endogenous alpha frequency after the cessation of stimulation. Thus it appears that IAPF is relatively stable and trait-like^31^, yet may be transiently manipulated.

One limitation of the current study is that we examined IAPF across the scalp explicitly in the context of a perceptual cycle hypothesis^43^, though IAPF and other oscillations may have novel functions in different cortical regions^79^. Future work will be required to determine whether IAPF may support different roles. The current findings were also limited in that we only examined relationships between IAPF during rest and cognition. Future research will be required to better understand whether IAPF is similarly impaired during cognitive engagement in schizophrenia, as well as its causal role in cognitive dysfunction. For example, it is unclear whether disruptions in perception in schizophrenia could be the result of fluctuations in the induced frequency and power of alpha prior to encoding a stimulus, or perhaps more specific aspects of alpha phase coherence and timing that are predictive of optimal encoding windows. Additionally, the current investigation was limited in that it only compared IAPF between patients with schizophrenia against healthy controls. Given similar IAPF abnormalities observed across other forms of psychopathology and aging, abnormal alpha cycling may reflect a more generalized phenomenon related to impaired cognition. Finally, the current study did not thoroughly examine relationships between IAPF and power dynamics across the time–frequency spectrum, though we note that slower IAPF may correspond to higher theta power in schizophrenia, which we observe in this sample.

To summarize, we found that IAPF was reduced in schizophrenia during rest, related to deficits in both visual attention and global cognition, and that disruptions in sustained visual attention discrimination mediated the relationship between IAPF and global cognition across groups. These findings suggest that slower alpha cycles reflect disruptions in perceptual sensitivity that may account for generalized cognitive deficits observed in schizophrenia. Future studies will be required to determine whether these relationships are causal, and whether they reflect specific aspects of pathophysiology related to schizophrenia or more directly relate to cognitive dysfunction. Critically, the current findings suggest that IAPF may serve as a promising treatment target for neuromodulation and other neuroplasticity-based interventions directed at improving cognition.

## Supplementary Information

Supplementary Information.
