# Author Correction: Individual alpha peak frequency is slower in schizophrenia and related to deficits in visual perception and cognition

**DOI:** 10.1038/s41598-021-00055-6

**Published:** 2021-10-11

**Authors:** Ian S. Ramsay, Peter A. Lynn, Brandon Schermitzler, Scott R. Sponheim

**Affiliations:** 1 Department of Psychiatry & Behavioral Sciences, University of Minnesota, Minneapolis, MN USA; 2 Minneapolis Veterans Affairs Health Care System, Minneapolis, MN USA

Correction to: *Scientific Reports* 10.1038/s41598-021-97303-6, published online 08 September 2021

The original version of this Article contained errors in the spelling of the authors Peter A. Lynn and Scott R. Sponheim which were give as Peter Lynn and Scott Sponheim respectively.

The original Article has been corrected.

