# Supplementary material for: Individual alpha peak frequency is slower in schizophrenia and related to deficits in visual perception and cognition
Source: Sci Rep. 2021 Sep 8;11:17852. doi: 10.1038/s41598-021-97303-6 (PMC8426382; doi:10.1038/s41598-021-97303-6)
Supplement: Supplementary file 1 — Supplementary Information. [file 41598_2021_97303_MOESM1_ESM.docx]

**Supplementary Figures**

Figure S1. log-log Power Spectrum


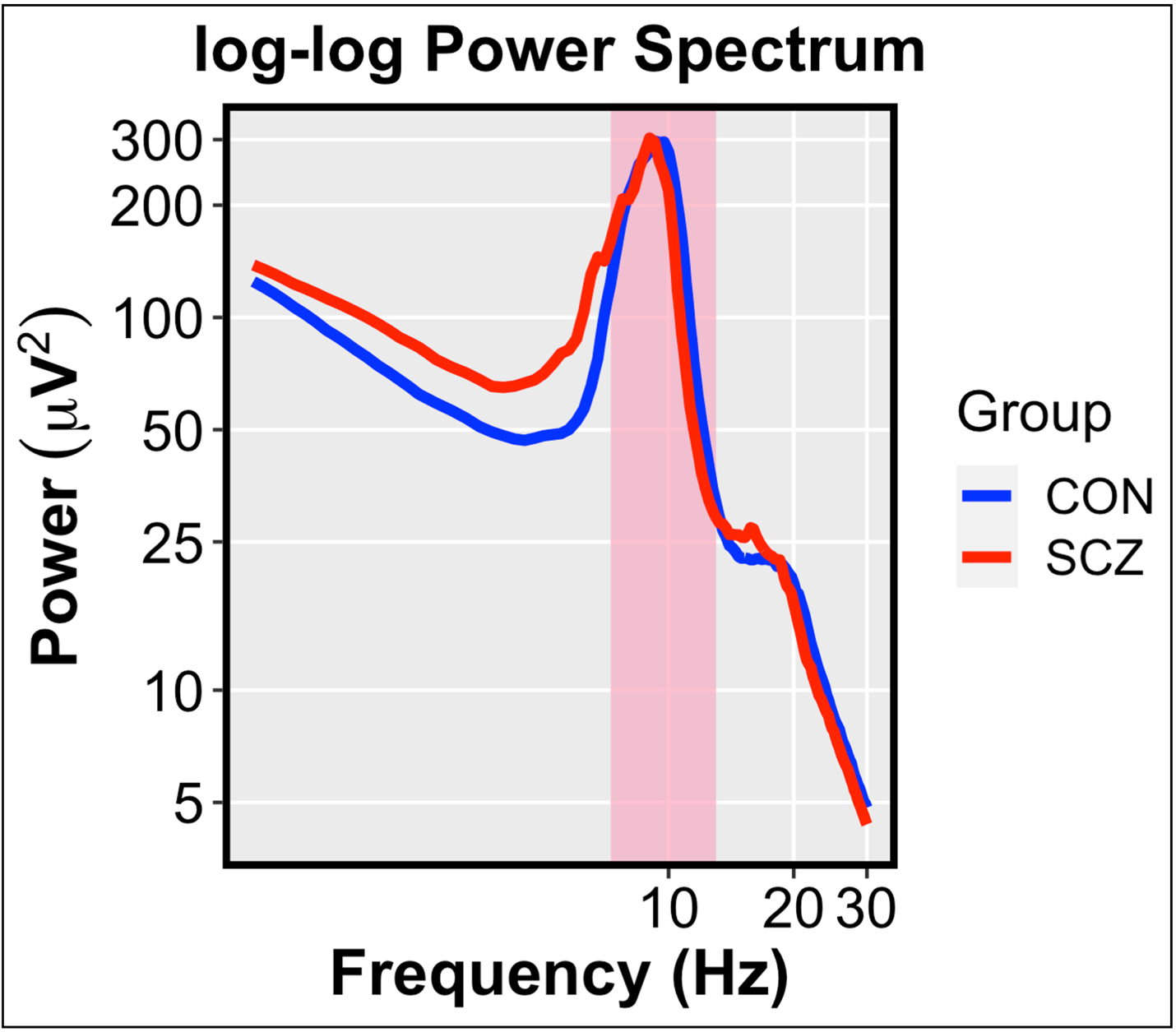


Figure S2. Distribution of IAPF


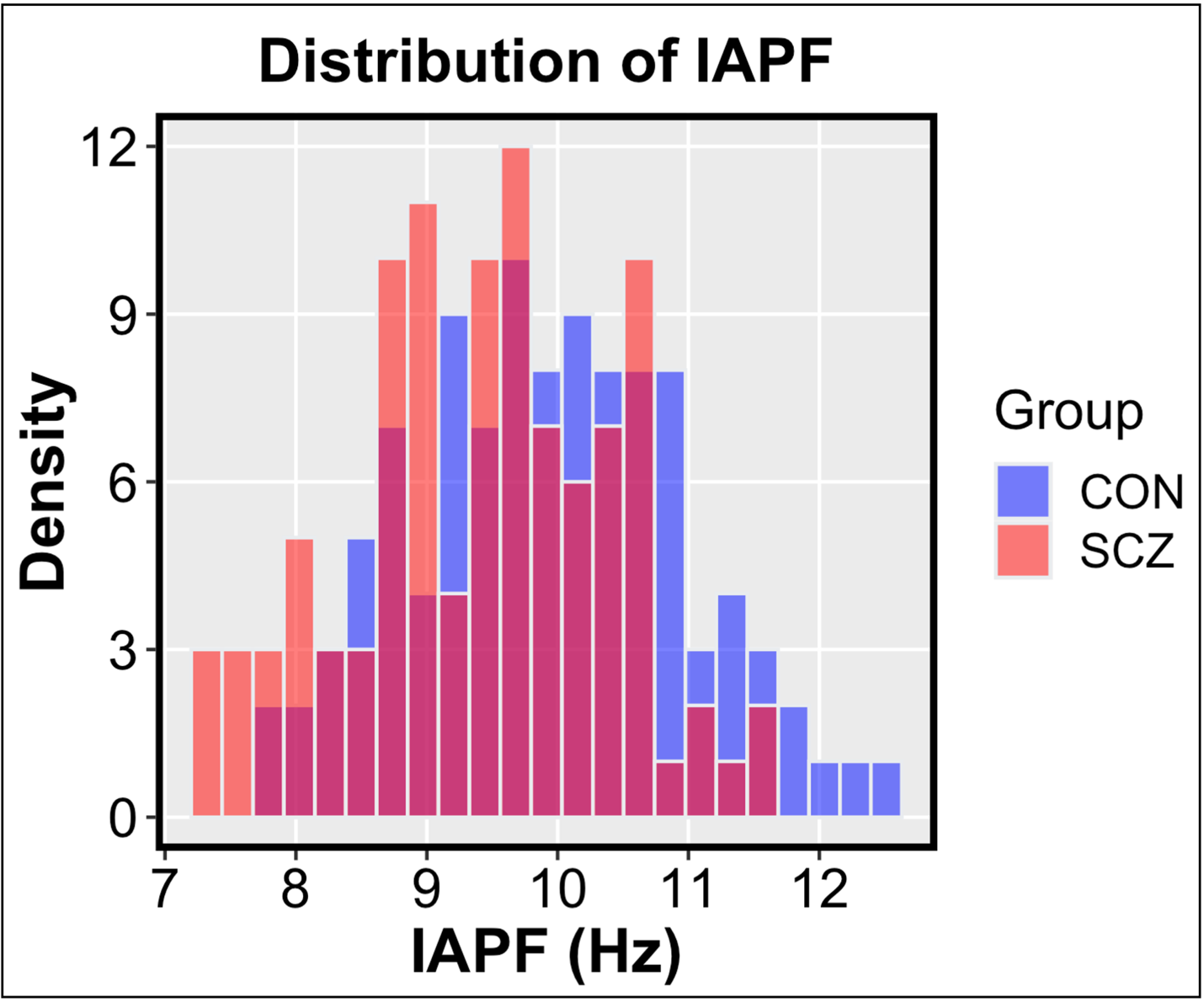


Figure S3. Individual Electrode IAPF for SCZ, CON, and Group Average


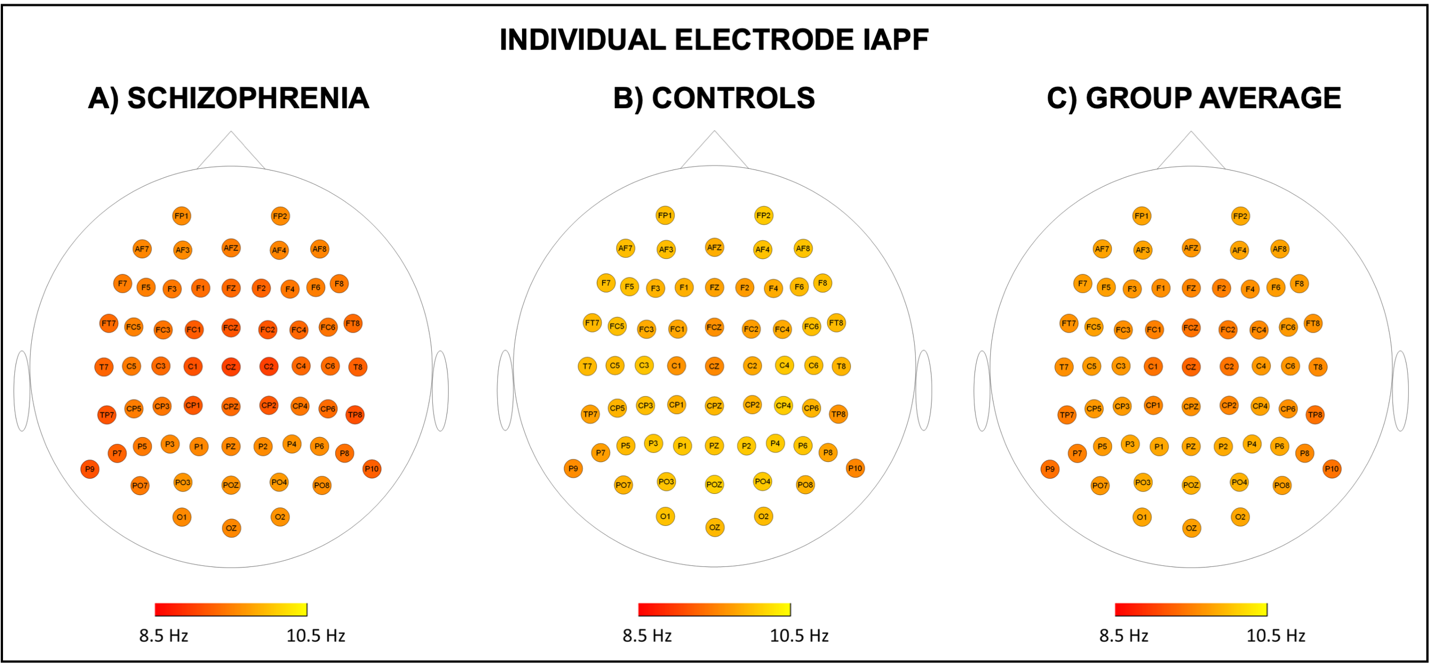


**Supplementary Tables**

| **Table S1: Between Group ANCOVA** | | |  |
| --- | --- | --- | --- |
|  | t-value | P-value |  |
| Group | 14.73 | 0.0002 |  |
| Age | 10.91 | 0.001 |  |
| Gender | 3.01 | 0.08 |  |
| Study Montage | 1.01 | 0.37 |  |
|  |  |  |  |
| **Table S2: Linear Model: DS-CPT d' ~ IAPF** | | | |
|  | t-value | P-value |  |
| IAPF | 3.45 | 0.0007 |  |
| Age | 1.66 | 0.1 |  |
| Gender | 0.02 | 0.98 |  |
| Study Montage 1 | -0.83 | 0.41 |  |
| Study Montage 2 | 1.08 | 0.28 |  |
| Group x IAPF | -0.602 | 0.55 |  |
|  |  |  |  |
| **Table S3: Linear Model: Global Cognition ~ IAPF** | | | |
|  | t-value | P-value |  |
| IAPF | 3.04 | 0.003 |  |
| Age | 1.98 | 0.049 |  |
| Gender | 2.78 | 0.006 |  |
| Study Montage 1 | 1.09 | 0.28 |  |
| Study Montage 2 | -0.06 | 0.95 |  |
| Group x IAPF | -0.26 | 0.8 |  |
|  |  |  |  |
| **Table S4: Linear Model: Processing Speed ~ IAPF** | | | |
|  | t-value | P-value |  |
| IAPF | 2.94 | 0.004 |  |
| Age | -2.06 | 0.04 |  |
| Gender | 5.23 | 5E-07 |  |
| Study Montage 1 | 0.59 | 0.56 |  |
| Study Montage 2 | -0.93 | 0.35 |  |
| Group x IAPF | 1.03 | 0.3 |  |
|  |  |  |  |
| **Table S5: Linear Model: Perceptual Reasoning ~ IAPF** | | | |
|  | t-value | P-value |  |
| IAPF | 4.12 | 0.00006 |  |
| Age | -2.16 | 0.03 |  |
| Gender | 0.82 | 0.41 |  |
| Study Montage 1 | -0.4 | 0.69 |  |
| Study Montage 2 | 0.04 | 0.97 |  |
| Group x IAPF | 0.05 | 0.96 |  |
|  |  |  |  |
| **Table S6: Linear Model: Working Memory ~ IAPF** | | | |
|  | t-value | P-value |  |
| IAPF | 2.23 | 0.03 |  |
| Age | 0.4 | 0.69 |  |
| Gender | 0.57 | 0.57 |  |
| Study Montage 1 | 0.37 | 0.71 |  |
| Study Montage 2 | -0.75 | 0.45 |  |
| Group x IAPF | 0.44 | 0.66 |  |
|  |  |  |  |
| **Table S7: Linear Model: Verbal Reasoning ~ IAPF** | | | |
|  | t-value | P-value |  |
| IAPF | 2.53 | 0.01 |  |
| Age | 3.43 | 0.0007 |  |
| Gender | 0.68 | 0.5 |  |
| Study Montage 1 | 1.61 | 0.11 |  |
| Study Montage 2 | 1.44 | 0.15 |  |
| Group x IAPF | -2.21 | 0.03 |  |
|  |  |  |  |
